# C-terminal-truncated HBV X promotes hepato-oncogenesis through inhibition of tumor-suppressive β-catenin/BAMBI signaling

**DOI:** 10.1038/emm.2016.107

**Published:** 2016-12-02

**Authors:** Seok Lee, Mi-Jin Lee, Jun Zhang, Goung-Ran Yu, Dae-Ghon Kim

**Affiliations:** 1 Division of Gastroenterology and Hepatology, Department of Internal Medicine, The Research Institute of Clinical Medicine of Chonbuk National University, Jeonju, Jeonbuk, Republic of Korea; 2 Biomedical Research Institute of Chonbuk National University Hospital, Jeonju, Jeonbuk, Republic of Korea

## Abstract

C-terminal-truncated hepatitis B virus (HBV) X (HBx) (ctHBX) is frequently detected in hepatocellular carcinoma (HCC) through HBV integration into the host genome. However, the molecular mechanisms underlying ctHBx-associated oncogenic signaling have not yet been clarified. To elucidate the biological role of ctHBx in hepato-oncogenesis, we functionally analyzed ctHBx-mediated regulation of the activin membrane-bound inhibitor bone morphogenetic protein and activin membrane-bound inhibitor (BAMBI) through transforming growth factor-β (TGF-β) or β-catenin (CTNNB1) in HCC cells and in an animal model, and we compared its role to that of the full-length HBx protein. Ectopic ctHBx expression generated more colonies in anchorage-dependent and -independent growth assays than did HBx expression alone. ctHBx downregulated BAMBI to a greater degree than did HBx in HCC cells. HBx activated the Wnt/β-catenin pathway, which positively regulated the BAMBI expression through T-cell factor 1 signaling, whereas ctHBx negatively regulated the Wnt/β-catenin pathway. BAMBI downregulated the β-catenin and TGF-β1 signaling pathways. TGF-β1 positively regulated BAMBI expression thorough Smad3 signaling. Furthermore, knockdown of BAMBI was more tumorigenic in HCC cells. Therefore, downregulation of both β-catenin and TGF-β1 signaling by BAMBI might contribute to tumor suppression in mice xenotransplanted with HepG2 or SH-J1 cells. Taken together, ctHBx may have a more oncogenic role than HBx through its inhibition of tumor-suppressive β-catenin/BAMBI signaling.

## Introduction

Hepatocellular carcinoma (HCC) is a highly malignant tumor type and is the third most frequent cause of cancer-related death in the United States and Europe.^1^ A strong epidemiological correlation between chronic hepatitis B virus (HBV) infection and HCC occurrence has long been apparent.^2^ The HBV genome is a partially double-stranded circular DNA that is 3.2 kb in length and encodes genes for the S, X, C and P proteins. Among the four proteins translated by HBV, the hepatitis B virus X (HBx) oncoprotein has been implicated in HBV-mediated hepato-carcinogenesis.^3, 4^ During hepato-carcinogenesis, cancer cells gain an advantage through the selective reduction of the tumor-suppressive activity of transforming growth factor-β (TGF-β), together with augmentation of its oncogenic activity.^5^ Furthermore, HBx has been reported to shift human TGF-β signaling from tumor suppression to oncogenesis in early chronic hepatitis B.^6^ Therefore, alterations in the TGF-β signal transduction pathway may be involved in HCC development. HBx mutants, particularly C-terminal-truncated mutants (ctHBx), are frequently detected in the tumor tissues of HCC patients but are rarely observed in surrounding non-tumor tissues.^7, 8^ Furthermore, these mutant forms of HBx can also enhance the transformation abilities of *ras* and *myc.*^9^ However, little is known regarding whether or how ctHBx might be involved in carcinogenesis and tumor progression, especially in association with bone morphogenetic protein (BMP) and activin membrane-bound inhibitor (BAMBI)/TGF-β signaling in hepato-carcinogenesis. The assumption that ctHBx is oncogenic through altered BAMBI/TGF-β signaling was the basis for this study.

BAMBI, a transmembrane glycoprotein evolutionally conserved in species ranging from *Xenopus* to *Homo sapiens*, is highly homologous to TGF-β/BMP type-1 receptors (TGFR1/BMPR1), except that it lacks an intracellular serine/threonine kinase domain. BAMBI is incorporated into complexes with TGF-β/BMP/activin type-2 receptors (TGFR2/BMPR2) and, as a pseudoreceptor, antagonizes all TGF-β/BMP and activin signaling.^10, 11^ BAMBI is tightly co-expressed with BMP4 during embryonic development in zebrafish, *Xenopus*, birds and mice^10, 12, 13, 14^ through TGF-β^15^ and Wnt signaling.^16^

Human BAMBI can also promote Wnt signaling by enhancing its interaction with the receptor Frizzled-5.^17^ Its elevated expression has been suggested to attenuate TGF-β-mediated growth arrest in colorectal and HCC cells^16^ as well as induce the growth and invasion of human gastric carcinoma cells.^18^ BAMBI expression is also associated with Toll-like receptor 4- and lipopolysaccharide-mediated hepatic fibrosis.^19^ Therefore, we first assessed the relationship between ctHBx or HBx and BAMBI/β-catenin signaling. Finally, we investigated the tumor-suppressive role of BAMBI in hepato-oncogenesis.

## Materials and methods

### Ethics statement

All patients provided informed consent for a protocol that was reviewed and approved by the institutional review board of Chonbuk National University Hospital. Written informed consent was obtained from all patients. The study using experimental animals was approved by the Institutional Animal Care and Use Committee at Chonbuk National University (CUH-IACUC-140121-12).

### Suppression subtractive hybridization (SSH)

SSH was performed between paired HCC and non-HCC tissue with the Clontech PCR-Select cDNA Subtraction Kit (Clontech, Mountain View, CA, USA) according to the manufacturer's protocol for HBV-associated HCC patients using two-way subtraction. In the forward subtraction, the codes from HCC were used as the tester, and the codes from paired non-HCC tissues were used as the driver; in the reverse subtraction, the codes from the paired non-HCC tissues were used as the tester, and the codes from HCC were used as the driver. Ten nanograms of the PCR product was cloned into the pGEM-T Easy Vector plasmid (Promega, Madison, WI, USA) and transformed into competent *E. coli* XL2-Blue cells (Stratagene, Cedar Creek, TX, USA). Two thousand colonies were randomly selected. The plasmids were purified on Qiaprep Spin Columns (Qiagen, Hilden, Germany), and the insert sequences were determined by restriction endonuclease digestion with *Eco*RI. The final sequence homolog searches were performed using the GenBank (nr) and EST (dbEST) databases employing the BLASTN algorithm at the National Center for Biotechnology Information (http://www.ncbi.nlm.nih.gov/BLAST).

### Semiquantitative reverse transcriptase-PCR (RT-PCR)

Genomic DNA was isolated from 50 pairs of HCCs from patients with positive serum hepatitis B surface (HBs) antigen. For PCR amplification of HBx, sets of PCR primers (44 F: 5′-TCCTTTGTTTACGTCCCGTC-3′, 210 R: 5′-CGTTCACGGTGGTCTCCAT-3′ and 465 R: 5′-TTAGGCAGAGGTGAAAAAGTTGC-3′) were used for full-length and COOH-truncated HBx, respectively.

### Cloning of Enh1/Xp-ctHBx and ctHBx

Initially, we obtained an EST clone that contained a partial HBx cDNA fused to a cDNA for the 5′ side of dual specificity phosphatase 26 in the chromosome 8 genomic contig. The HBx cDNA was truncated at the C-terminus by the stop codon at the junction of two genes, suggesting that a ctHBx was produced through HBV integration into the DNA and was functionally active in HCC. The cloning of full-length cDNAs of *Enh1/Xp-tHBx* was completed through two cycles of RT-PCR and overlap primer extension after cDNA amplification of the mRNA. First, *ctHBx* was extended by searching the human HBV database; the extended sequence was confirmed using RT-PCR (primers: 5′-CACCTCCTTCCCATGGCT-3′ and 5′-CACTTCCCCTGTAATCCC-3′), and it contains the initiation codon. Next, *Enh1/Xp* was PCR-amplified using the specific primers (5′-GRAAAYTGCCTGTAAAT-3′ and 5′-GGGTCGTCCGCGGGATTCAG-3′). Finally, full-length *Enh1/Xp-ctHBx* was obtained by overlapping primer extension using primers containing *Kpn*I and *Xho*I restriction enzyme sites (5′-cggggtaccGRAAAYTGCCTGTAAAT-3′ and 5′-ccgctcgagCACTTCCCCTGTAATCCC-3′), respectively, and was then ligated into pGEMT-Easy Vector (Promega). The full-length ctHBx gene was synthesized using PCR and specific primers for *HBx*: 5′-ccggaattcCCTTCCCATGGCTG-3′ (forward) and 5′-ccgctcgagCACTTCCCCTGTAATCCC-3′ (reverse). The reaction conditions were 94 °C for 5 min; 40 cycles of 94 °C for 1 min, 45–55 °C for 1 min, 72 °C for 1 min; and 72 °C for 7 min.

### Luciferase reporter gene assay

Transcriptional activation assays for the *BAMBI* promoter (−255 to +82 bp) and its mutants (mt-All-Luc: mutation of all three Smad-binding element (SBE) CAG(A/C)C sequences)^11^ and for the *TGF-β1* promoter (−1362 to +11) and its mutant (mutation of the 3′ motif of TGF-β1-DR1)^20^ were conducted using luciferase reporters driven by the respective promoters. T-cell factor (TCF)-1/4-dependent transcriptional activation was also examined through a luciferase reporter gene assay using pTOP-Flash and its mutant pFOP-Flash. The dual luciferase assay was performed according to the manufacturer's instructions (Promega).

### Immunoblotting

A total of 30 μg of each cell or tissue lysate was separated by sodium dodecyl sulfate-polyacrylamide gel electrophoresis, and the proteins were subsequently transferred to Hybond membranes (Amersham Pharmacia Biotech, Buckinghamshire, UK). The membranes were blocked and then incubated with the designated primary antibodies, and signals were detected using an ECL Western Blotting Kit (Amersham Pharmacia Biotech). Goat anti-BAMBI polyclonal antibody (R&D Systems, Minneapolis, MN, USA), anti-TGF-β1 (Santa Cruz Biotechnology, Inc., Santa Cruz, CA, USA) anti-β-catenin (BD Biosciences, Franklin Lakes, NJ, USA), anti-HBx (Chemicon International, Temecula, CA, USA) and β-actin (Sigma, Saint Louis, MO, USA) were used as the primary antibodies.

### Immunofluorescence

HepG2 and Hep3B cells were grown on glass coverslips, fixed in 4% paraformaldehyde and permeabilized with 0.2% Triton X-100 in phosphate-buffered saline. Coverslips were incubated for 1 h in phosphate-buffered saline containing 0.1% bovine serum albumin and a mixture of antibodies. After two washes in phosphate-buffered saline, the cells were incubated for 1 h with TRITC-conjugated anti-rabbit antibody (1:100 dilutions) (DAKO, Glostrup, Denmark). DNA was labeled using the Hoechst 33258 dye (1 μg ml^−1^). Cells were examined using laser scanning microscopy (LCM 510; Carl Zeiss, Jena, Germany) as previously described.^21^

### Anchorage-independent growth

For the anchorage-independent growth assay, 1 × 10^5^ cells were plated in 0.3% low melting point agar/growth medium onto 6-cm dishes with a 0.6% agar underlay. After 4 weeks, colonies that were >1 mm in diameter were counted.

### Preparation of the recombinant adenovirus

To prepare the BAMBI-expressing adenovirus, a GFP-tagged BAMBI cDNA fragment was cloned into the *Kpn*I and *Not*I sites of pENTR2B (Invitrogen, Carlsbad, CA, USA), and the GFP-tagged BAMBI insert was transferred to the pAd/CMV/V5-DEST vector (Invitrogen) by the Gateway system using LR Clonase (Invitrogen). Plasmids were linearized with *Pac*I (Promega) and transfected into 293 A cells using Lipofectamine 2000 transfection reagent. The resulting 293A cells were cultured for 1–2 weeks in Dulbecco's modified Eagle's medium containing 10% fetal bovine serum, and the medium was replaced every 2 days. As a control, the pAd/CMV/V5-GW/lacZ vector (Invitrogen) was used to produce lacZ-bearing adenovirus.

### Bioluminescence imaging and analysis

We established an orthotopic nude mouse subcutaneous tumor model with SH-J1-Luc cells overexpressing BAMBI by adenovirus delivery. Briefly, 3 × 10^6^ cells in 0.08 ml of culture medium and 0.04 ml of 10% Matrigel were injected into the subcutaneous tissues of both shoulders of Balb/c nude mice (4 weeks old, *n*=5). Tumor growth was monitored once a week for 4 weeks using the Xenogen IVIS 100 Imaging System (Caliper Lifescience, Hopkinton, MA, USA; 1 min, Level B/FOV15). Mice were anesthetized with 3% isofluorane after administration of 150 mg kg^−1^ firefly D-luciferin (Caliper Lifescience) via intraperitoneal injection for imaging.

### Short hairpin RNA (shRNA)-expressing lentiviral vectors and transduction

Lentivirus vectors encoding a shRNA targeting BAMBI, β-catenin or TGFβ1 (TRCN0000330319 target sequence:

CCGGCTTGATCCTCAGAACTCAAATCTCGAGATTTGAGTTCTGAGGATCAAGTTTTTG, TRCN0000314991 target sequence:

CCGGTTGTTATCAGAGGACTAAATACTCGAGTATTTAGTCCTCTGATAACAATTTTTG and the TRCN0000318388 target sequence:

CCGGCAAGCAGAGTACACACAGCATCTCGAGATGCTGTGTGTACTCTGCTTGTTTTTG) as well as the shRNA non-specific control (SHC002V) were used for transduction of HepG2 cells according to the manufacturer's instructions (Sigma Chemical Co). In brief, 5 × 10^4^ cells were incubated overnight in a 24-well plate and then transduced with lentiviral particles at a multiplicity of infection of 1 in the presence of 8 μg ml^−1^ hexadimethrine bromide. Western blotting analysis was performed to confirm specific knockdown of each vector.

### Statistical analysis

The data are expressed as the mean±s.d. Differences were analyzed using dependent or independent *t*-tests. Values of *P*<0.05 were considered significant.

## Results

### SSH and integrated HBx expression

Clinical characteristics of tumor and non-tumor tissues were summarized in two patients with HCC (Supplementary Table 1). All immunohistological findings were consistent with the serological findings. Next we used SSH to monitor changes in gene expression in HCC tissues and non-tumor tissues from Case 1, as previously described.^22, 23^ To select genes associated with HCC and functional HBx, a total of approximately 2,000 subtracted cDNA fragments were obtained from a two-way (forward and reverse) subtraction library. We identified candidate genes based on more than four appearances (Supplementary Tables 2 and 3). The digested HBV-DNA patterns showed integration of HBV-DNA in both tumor and non-tumor tissues in Case 1 (data not shown). However, the integration of HBV-DNA was not observed in tumor or non-tumor tissue in Case 2. Northern blotting analysis revealed that *HBx* and the middle of *HBs* mRNA were expressed in HCC (tumor) tissue but were only minimally observed in the non-tumor tissue in Case 1 (data not shown), in which an integrated stage of chronic HBV infection was observed (low HBV-DNA and positive anti-HBe Ab). In contrast, neither *HBx* nor the middle of *HBs* was expressed in the HCC or non-tumor tissues in Case 2. Among the candidate genes, we were interested in the *BAMBI* expressed at a low frequency in HCC because it is a negative regulator of *TGF-β1*.^13, 14^

### Modulation of BAMBI expression by ctHBx

**Figure 1:**
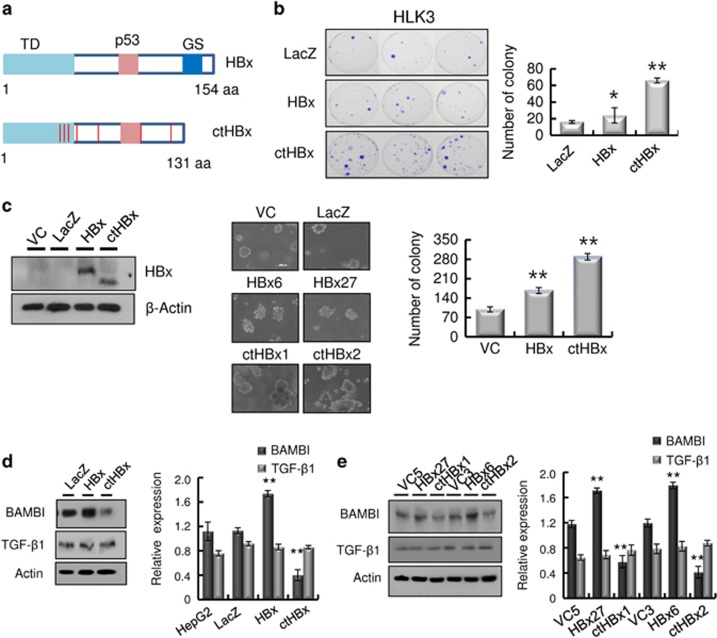
Regulation of BAMBI by ctHBx in HCC. (**a**) Putative amino-acid sequences of ctHBx. Point mutations and C-terminal deletion mutation of HBx (ctHBx) compared with those of wild-type HBx. TD, transforming domain (aa 1–50); p53, p53-mediated repression domain (aa 88−97); GS, growth-suppressive effect domain (aa 142–154). Red vertical lines represent mutations (lower panel). (**b**) Clonogenicities in the HLK3 cells after transfection with an expression vector for HBx or ctHBx, assessed according to the plating efficiency in a colony-forming assay. The number of colonies was visualized by crystal violet staining of the cell cultures after 14 days of G418 selection (left). Quantification of the number of colonies (right). Values represent the mean±s.d. from three independent experiments. *P<0.05; **P<0.01. (**c**) Different molecular sizes of HBx and ctHBx proteins in cell lysates of HLK3 cells transiently transfected with expression plasmids (left). HBx- or ctHBx-expressing cells were grown as colonies in soft agar, and the results were compared with those from the vector control (middle). The colonies shown are 15 days old. Quantification was performed in triplicate (right). The values represent the mean±s.d. from three independent experiments. **P<0.01. (**d**) Immunoblot analysis of BAMBI and TGF-β1 protein expression in the transient transfectants expressing HBx or ctHBx in HLK3 cells (left). Quantification of the relative protein expression (right). Values represent the mean±s.d. from three independent experiments. **P<0.01. (**e**) Immunoblot analysis of BAMBI and TGF-β1 protein expression in stable HepG2 transfectants expressing HBx or ctHBx (left). Quantification of the relative protein expression (right). Values represent the mean±s.d. from three independent experiments. **P<0.01.

HBx was detected in 50 HCC samples from 50 patients with positive HBs antigen, using PCR primers that covered 44–210 nt. However, HBx with an intact C-terminus was detected in 30 of the HCC samples (60%), using PCR primers that covered 44–465 nt (C-terminal primer set). The remaining 20 (40%) HCC samples without full-length HBx showed ctHBx (Supplementary Figure 1). We identified EST-F81, in which a C-terminal-truncated *HBx* gene was fused to the 5′ end of dual specific phosphatase 26 from the SSH EST library (Supplementary Figure 2a). Based on this clone, we isolated and analyzed the mutations in *Enh1/Xp* and *ctHBx* genes from the HCC tissues of Case 1 (Supplementary Figure 2b). *Enh1/Xp* contained seven nucleotide mutations, and the open reading frame of *HBx* included 131 amino acids (aa) and seven missense mutations (Supplementary Figure 3). Notably, this ctHBX contained three missense mutations in the transforming domain (aa 1−50) and a deletion of the growth-suppressive effect domain (aa 142−154) (Figure 1a). Consistently, colony-generation assays revealed that *ctHBx* produced greater numbers of colonies than *HBx* or the vector control in HepG2 cells (Figure 1b), in which the β-catenin gene was known to be mutated.^24^ We also found that the HBx and ctHBx proteins were successfully expressed in the selected colonies (Figure 1c, left). The capacities of HBx and ctHBx for anchorage-independent cell growth were investigated by determining the level of cell growth as indicated by the number of colonies observed in soft agar (Figure 1c, middle and right). Similarly, *ctHBx* produced many more colonies of HepG2 cells compared with *HBx* or the vector control. HepG2 cells with transiently or stably expressed ctHBx exhibited inhibition of BAMBI protein expression but no change in TGF-β1 protein expression (Figure 1d and e). In contrast, the HBx-expressing HepG2 cells showed a significant increase in BAMBI expression but no change in TGF-β1 expression. Real-time RT-PCR showed that ctHBx profoundly inhibited *BAMBI* and *TGF-β1* mRNA expression compared with HBx (Supplementary Figure 4). Therefore, these results suggest that BAMBI was transcriptionally downregulated by either HBx or ctHBx, but it was posttranscriptionally overexpressed through an HBx-mediated alternative pathway. In addition, TGF-β1 was transcriptionally downregulated by either HBx or ctHBx. However, its protein expression did not change much owing to low basal activity of the TGF-β1 promoter.

### Activation of β-catenin signaling and BAMBI upregulation by HBx, not by ctHBx

**Figure 2:**
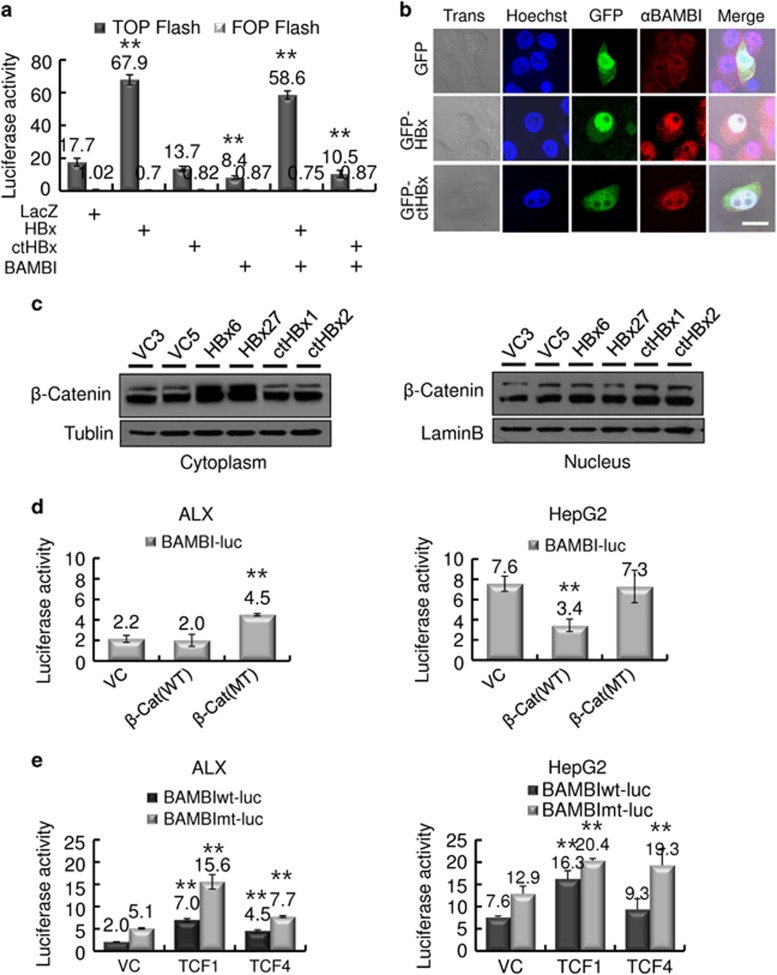
Modulation of Wnt/β-catenin activation by HBx, ctHBx or BAMBI. (**a**) HepG2 cells were transfected with either mock (LacZ), HBx, ctHBx or BAMBI plasmids alone or in combination with pTOP-Flash and pFOP-Flash reporter plasmids. After 48 h of culture, luciferase activity was measured. The values represent the mean±s.d. from three independent experiments. *P<0.01. (**b**) Subcellular localization of HBx and ctHBx proteins. HepG2 cells were transiently transfected with GFP-tagged HBx or ctHBx, fixed in 0.4% paraformaldehyde and subsequently processed for indirect immunofluorescence with antibodies against BAMBI (TRITC, red). Nuclei were stained with Hoechst 33258 (blue), and the cells were subsequently examined using confocal microscopy (Trans, transmission; bar, 20 μm). (**c**) Subcellular localization of β-catenin by HBx or ctHBx. β-Catenin expression was determined via immunoblot analysis in nuclear or cytoplasmic fractions of the stable transfectants expressing HBx or ctHBx. (**d**) Increased *BAMBI* promoter activity induced by mutant β-catenin. ALX and HepG2 cells were transfected with either wild-type or mutant β-catenin expression plasmids. After 48 h of culture, luciferase activity was measured. The values represent the mean±s.d. from three independent experiments. **P<0.01. (**e**) Modulation of wild-type or mutant *BAMBI* promoter activity by TCF-1 or TCF-4 in ALX and HepG2 cells. The values represent the mean±s.d. from three independent experiments. **P<0.01.

The effects of HBx, ctHBx and BAMBI on Wnt/β-catenin signaling were examined using a reporter plasmid containing multimerized TCF-4-binding sites linked to the luciferase gene. BAMBI efficiently inhibited TCF-4-dependent transcriptional activation, while HBx increased this activity, and ctHBx had no effect (Figure 2a). To determine the mechanisms responsible for TCF regulation, we analyzed the subcellular localization of HBx and ctHBx in HepG2 cells. Immunofluorescence assays revealed that endogenous BAMBI localized prominently in the cytoplasmic membrane and less so in the nuclei of the cells, while ectopic BAMBI was expressed in the cytoplasmic membrane of the cells (Supplementary Figure 5). Both HBx and ctHBx are abundantly localized in the nucleus and less in the cytoplasm of the cells (Figure 2b). BAMBI substantially colocalized with HBx and ctHBx. An immunoblot analysis showed that β-catenin accumulated in the cytoplasmic fraction in HBx transfectants, but less accumulation was identified in ctHBx transfectants (Figure 2c). These results suggest that cytoplasmic HBx appears to modulate Wnt/β-catenin signaling.

We also tested the effect of Wnt/β-catenin signaling on the regulation of *BAMBI* transcription. Mutant β-catenin increased the promoter activity of *BAMBI* by more than twofold in ALX (Alexander; PLC/PRF/5) cells with wild-type β-catenin, whereas wild-type β-catenin decreased this promoter activity in HepG2 cells containing mutant β-catenin (Figure 2d). These results suggest that the basal activity of BAMBI is high owing to increased β*-*catenin activity; therefore, the ectopic expression of wild-type β*-*catenin seems to decrease the basal activity of BAMBI by competitive inhibition. Next we investigated which TCF is associated with β-catenin-mediated BAMBI expression using a luciferase reporter containing a *BAMBI* promoter and its mutant that encoded mutated SBEs to exclude Smad-dependent *BAMBI* promoter activity. TCF-1 produced a greater increase in BAMBI promoter activity than did TCF-4 (Figure 2e). These results suggest that Wnt/β-catenin activation increases BAMBI expression via TCF-1.

### Regulation of BAMBI/TGF-β1 expression

**Figure 3:**
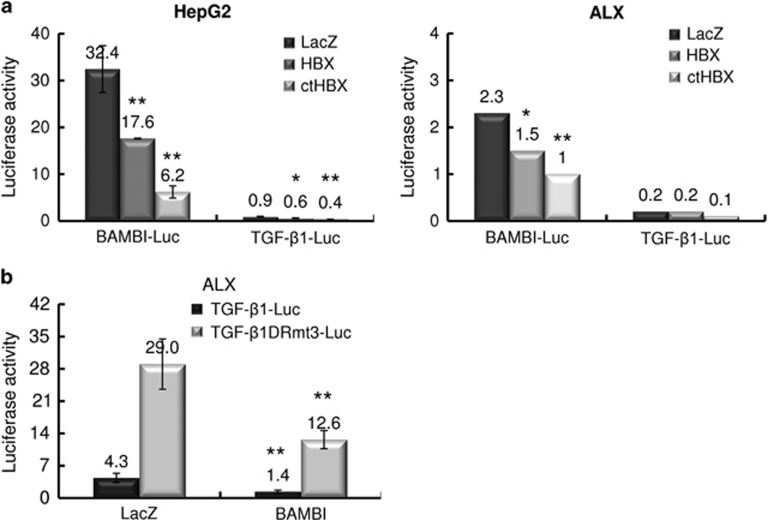
Modulation of *BAMBI/TGF-β1* expression by HBx or ctHBx. (**a**) HepG2 and ALX cells were transiently transfected with BAMBI or TGF-β1 reporter constructs along with the HBx or ctHBx expression plasmid. Values represent the mean±s.d. from three independent experiments (*P<0.05, **P<0.01). (**b**) ALX cells were transiently transfected with wild-type or mutant TGFβ1 reporter constructs (0.5 μg) along with the mock (LacZ) or BAMBI expression plasmid.

To evaluate the regulation of *BAMBI* promoter activity by HBx or ctHBx, we transiently co-transfected HepG2 or ALX cells with LacZ, HBx or ctHBx and a BAMBI-Luc reporter construct and measured the promoter activity (Figure 3a). Mock-transfected cells showed a high level of basal *BAMBI* promoter activity in Hep G2 cells, compared with ALX cells, in which the β-catenin gene was wild type.^24^ This finding suggests that β-catenin activation leads to BAMBI expression. Interestingly, ctHBx expression inhibited *BAMBI* promoter activity more profoundly than *HBx* expression. We first introduced a mutant form of the *TGF-β1* reporter construct (TGF-β1DRmt3), which contained a 3′ motif consisting of a direct repeat of the hexameric nucleotide sequence AGGTCA that is a peroxisome proliferator-activated receptor response element.^20^ Co-transfection of BAMBI resulted in a significant decrease in either wild-type or mutant *TGF-β1* promoter activity, although the basal promoter activity of wild-type TGF-β1 was low (Figure 3b). These results imply that BAMBI efficiently suppresses TGF-β1 signaling.

### Regulation of BAMBI by TGF-β1

**Figure 4:**
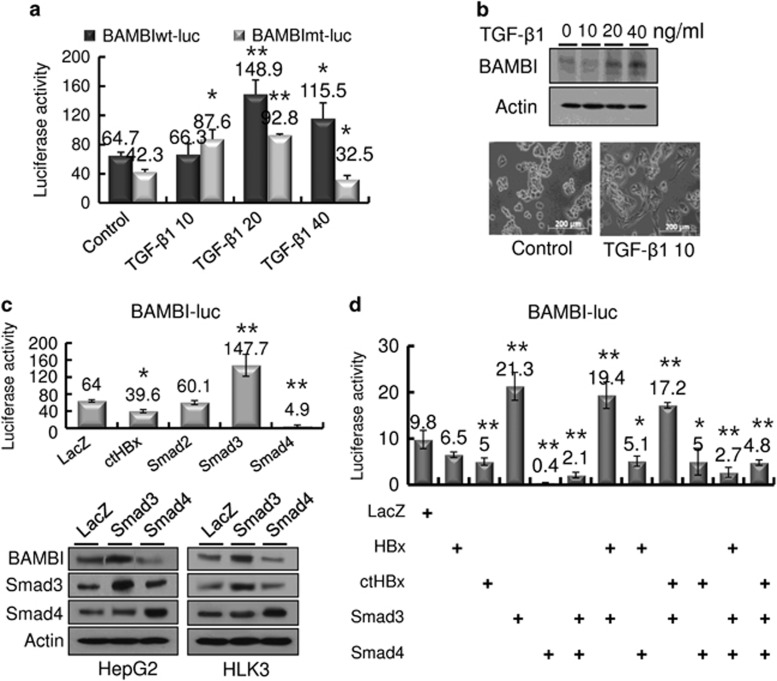
Regulation of BAMBI by TGF-β1. (**a**) Increased *BAMBI* promoter activity owing to exogenous TGF-β1 supplementation. HepG2 cells were transiently transfected with BAMBIwt-Luc (0.5 μg) or BAMBImt-Luc (0.5 μg) and treated with the indicated concentrations of TGF-β1 for 48 h. The values represent the mean±s.d. from three independent experiments. *P<0.05; **P<0.01. (**b**) Immunoblot analysis of BAMBI protein expression in HepG2 cells treated with TGF-β1 for 48 h (upper panels). Morphological changes in the HepG2 cells after treatment with exogenous TGF-β1 (lower panels). (**c**) Effects of the Smad family on *BAMBI* promoter activity (upper panel). Regulation of BAMBI protein expression by Smad3 or Smad4 (lower panels) in HepG2 and HLK3 cells. The values represent the mean±s.d. from three independent experiments. *P<0.05; **P<0.01. (**d**) Modulation of wild-type or mutant *BAMBI* promoter activity by ctHBx, Smad3 or Smad4, either alone or in combination. The values represent the mean±s.d. from three independent experiments. *P<0.05; **P<0.01.

Because BAMBI is known to be tightly co-expressed with BMP4 during embryonic development through TGF-β1^15^ and Wnt signaling,^16^ we next examined whether TGF-β1 regulates BAMBI expression. Exogenous treatment with TGF-β1 led to a dose-dependent increase in BAMBI promoter activity, with 20 ng ml^−1^ maximally stimulating *TGF-β1* promoter activity in HepG2 cells (Figure 4a). Similarly, the protein expression of BAMBI was enhanced through exogenous TGF-β1 treatment (Figure 4b, upper panels). Furthermore, TGF-β1 treatment induced a migratory and invasive morphology in the cells (Figure 4b, lower panels). To further dissect the TGF-β1-mediated BAMBI expression, we transfected HepG2 cells singly or in combination with ctHBX, Smad2, Smad3 or Smad4 (Figure 4c, upper). Smad3 substantially increased *BAMBI* promoter activity, whereas Smad4 decreased this activity. An immunoblot analysis revealed similar results (Figure 4c, lower panels). In the combination experiments, Smad3 overrode the ctHBx inhibition of *BAMBI* promoter activity (Figure 4d), while Smad4 overrode the stimulation of Smad3. ctHBx decreased *BAMBI* promoter activity more than HBx. These results suggest that TGF-β1-mediated BAMBI expression is dependent on Smad3 expression.

### Inhibition of tumorigenicity by BAMBI

**Figure 5:**
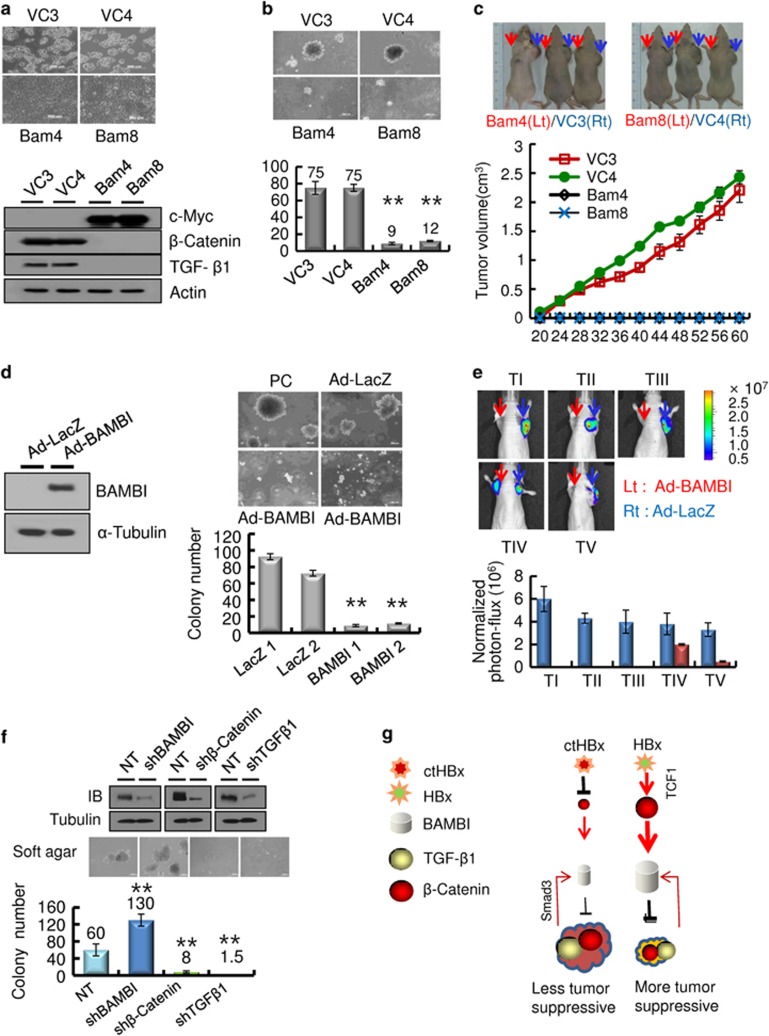
BAMBI functions as a tumor suppressor *in vitro* and *in vivo*. (**a**) BAMBI-mediated morphological changes in HepG2 cells stably overexpressing the BAMBI protein (upper panels). Immunoblot analysis of β-catenin and TGF-β1 in the stable transfectants overexpressing the c-Myc-tagged BAMBI protein (lower panels). (**b**) The BAMBI-expressing HepG2 cells showed minimal colony growth in soft agar compared with that of the vector control (upper panels). The colonies shown are 15 days old. Quantification was performed in triplicate (lower panel). The values represent the mean±s.d. from three independent experiments. **P<0.01. (**c**) Growth of the tumor masses in BAMBI-expressing HepG2 cells and vector control cells injected into the left shoulders (red arrows) of nude mice. Vector control cell lines injected into the right shoulder (blue arrows), grew quickly and formed tumor masses, whereas the BAMBI-expressing cells did not (upper panels). The tumor volume was measured as a function of time (lower panel). Each value represents the mean±s.d. (**d**) BAMBI expression in SH-J1 cells infected with either Ad-LacZ or Ad-BAMBI (left). The Ad-BAMBI-infected SH-J1 cells showed minimal colony growth in soft agar compared with the parent or Ad-LacZ-infected cells (right upper panels). The colonies shown are 15 days old. Quantification was performed in triplicate (right lower panel). The values represent the mean±s.d. of three independent experiments. **P<0.01. (**e**) Bioluminescent images of the subcutaneous tumor masses (*n*=5 mice per group, representative anterior–posterior images, 1 min exposure time). SH-J1 cells infected with Ad-LacZ subcutaneously injected into the right shoulder (blue arrows) grew quickly and formed tumor masses, but the Ad-BAMBI-infected cells injected into the left shoulder (red arrows) formed rare, small tumor masses (upper panels). Quantitative measurement of photon flux (lower panel). The values represent the mean±s.d. (**f**) Silencing of TGF-β1/BAMBI/β-catenin protein expression by lentiviral delivery of shRNA (upper panels) in HepG2 cells and their clonogenic ability in the soft agar (middle panels) and quantification (lower panel). The values represent the mean±s.d. of three independent experiments. **P<0.01. (**g**) A schematic model of the inhibition of BAMBI signaling by ctHBx. HBx profoundly stimulates Wnt/β-catenin signaling, and overexpression of BAMBI suppresses both TGF-β1 and β-catenin signaling and subsequently inhibits tumorigenicity. 
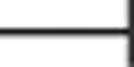
, inhibition; 
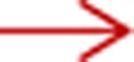
, stimulation.

Finally, we determined the functional role of BAMBI in HepG2 cells. The stable transfectants that expressed BAMBI showed morphological changes, including polygonal and epitheloid monolayer types (Figure 5a, upper panels). An immunoblot analysis of these transfectants showed that the expression of both β-catenin and TGF-β1 was profoundly suppressed (Figure 5a, lower panels). These stable transfectants proliferated only slightly and produced small-sized colonies on soft agar compared with those produced by vector control cells (Figure 5b). In the xenotransplant mouse model, the stable transfectants hardly formed tumor masses, whereas the vector controls almost always produced tumor masses (Figure 5c). These results imply that ectopic BAMBI functions as a tumor suppressor in HepG2 cells through the strong suppression of both β-catenin and TGF-β1. Alternatively, we examined whether BAMBI overexpression reduced tumorigenicity *in vivo*. A malignant HCC cell line (SH-J1) was transduced with either Ad-BAMBI/GFP or Ad-LacZ (Figure 5d, left), and colony-generation activity was examined on soft agar. SH-J1 transduced with Ad-BAMBI formed very few colonies, but control cells produced numerous colonies (Figure 5d, right lower panel). These transductants were subcutaneously inoculated into mice. Sufficient bioluminescence data were collected by 4 weeks (Figure 5e). SH-J1 cells transduced with Ad-LacZ developed large tumors >2000 mm^3^ in volume within 4 weeks. However, the growth of SH-J1 cells transduced with Ad-BAMBI was markedly suppressed (Supplementary Figure 6), suggesting that BAMBI overexpression leads to significant inhibition of tumor growth *in vivo*. Next knockdown in HepG2 cells was established by lentiviral delivery of BAMBI, β-catenin or TGF-β1 shRNAs (Figure 5f, upper panels). The colony-formation ability was compared with that of cells transduced with lentiviral non-target shRNA on the soft agar (Figure 5f, lower panel). BAMBI knockdown cells showed more colonies than non-target control cells. In contrast, the β-catenin or TGF-β1 knockdown cells produced fewer colonies than the non-target control cells. These results robustly support the possibility that ctHBx is more oncogenic than HBx through the inhibition of tumor-suppressive β-catenin/BAMBI signaling, which seems to be derived from a C-terminal truncation of HBx (Figure 5g).

## Discussion

BAMBI, a negative regulator of TGF-β activation, is thought to have a role in hepato-oncogenesis because the expression of BAMBI is aberrantly elevated in some HCCs relative to its expression in corresponding non-cancerous tissues.^16^ ctHBx mutants found in HCCs have been shown to confer a selective clonal advantage in preneoplastic or neoplastic hepatocytes through the abrogation of p53-mediated apoptosis, which contributes to hepatocellular carcinogenesis.^9, 25^ We speculated that functional ctHBx/HBx modulates the expression of BAMBI and TGF-β1, thereby influencing the development of HCC.

Our data consistently revealed that ctHBx overexpression enhanced the colony-forming ability and proliferative capacity in immortalized hepatocytes more than HBx overexpression. The transactivation activity of the C-terminal-deleted mutant of *HBx* was analyzed using a reporter construct of CRE-Luc and a luciferase assay, and the C-terminal region of HBx is crucial for transcriptional function.^9^ The specific transcriptional activity of *ctHBx* remains unclear; however, our data revealed that both HBx and ctHBx are abundantly localized in the nucleus and less in the cytoplasm of HepG2 and ALX cells. An immunoblot analysis showed that β-catenin accumulated in the cytoplasmic fraction in HBx transfectants but less in ctHBx transfectants. Cytoplasmic HBx appears to modulate Wnt/β-catenin signaling. In HepG2 cells, the basal level of BAMBI activation was relatively high, while that of TGF-β1 was low, as previously observed in other HCC cases.^10, 16^ BAMBI is a transmembrane protein that lacks an intracellular kinase domain but has high sequence similarity to the extracellular domain of TβRI and thus inhibits TGF-β signaling by forming a heterodimer with TβRII.^10^ However, our data revealed that BAMBI also transcriptionally regulates *TGF-β1* expression.

BAMBI is also directly induced by TGF-β signaling through three tandem repeats of a 13-bp sequence containing SBEs. In other words, BAMBI transcription is regulated through the direct binding of Smad3 and Smad4 to its promoter.^11^ However, our cell culture system showed that Smad3 stimulated *BAMBI* promoter activity, whereas Smad4 depressed it. This Smad3-mediated BAMBI expression can contribute to hepato-carcinogenesis through TGF-β signaling, as previously reported.^6^ Furthermore, a mutation in the SBE abrogated the Smad3-stimulatory effect but did not change the inhibitory activity of Smad4. Therefore, the SBE in BAMBI seems to be the binding site for Smad3, but not for Smad4.

β-Catenin mutation leads to the functional activation of Wnt/β-catenin signaling in hepatoma cells, and the amount of literature on the stabilizing mutation of β-catenin in HCC is increasing.^26, 27, 28^ In addition to mutations of β-catenin, ectopic expression of HBx along with Wnt-1 has also been reported to activate Wnt/β-catenin signaling in Huh7 cells by stabilizing cytoplasmic β-catenin, which was achieved by suppressing glycogen synthesis kinase 3 activity via the activation of Src kinase.^29^ Our study showed that HBx activated Wnt/β-catenin signaling, which in turn positively regulated BAMBI through TCF-1. BAMBI negatively modulated Wnt/β-catenin signaling in HepG2 HCC cells, which differs from previous data found in HEK293T cells.^17^ Although, β*-*catenin activation increased the promoter activity of BAMBI, the deletion mutants of ctHBx and HBx decreased the promoter activity of *BAMBI* in HepG2 and ALX cells because they have transactivation domains and regulate the transcriptional activity of other target genes in addition to β-catenin target genes.^30, 31^ ctHBx profoundly suppressed BAMBI promoter activity owing to the lack of activated Wnt/β-catenin signaling, whereas HBx less profoundly suppressed BAMBI promoter activity through activated Wnt/β-catenin signaling. Thus our data suggest that HBx stimulates Wnt/β-catenin signaling, which is followed by tumor-suppressive β-catenin/BAMBI signaling. Furthermore, ectopic overexpression of BAMBI negatively downregulated both TGF-β1 and β-catenin signaling in HepG2 cells, which consequently appeared to be associated with the suppression of oncogenicity. Thus the induction of BAMBI may function in part as a tumor-suppressor mechanism in HCC. In contrast, ctHBx seems to be more oncogenic than HBx through the inhibition of tumor-suppressive β-catenin/BAMBI signaling.

In conclusion, the data presented here provide a novel mechanism by which ctHBx might contribute to strong hepato-oncogenesis via the loss of tumor-suppressive β-catenin/BAMBI signaling.
