# Supplementary figures and images for: C-terminal-truncated HBV X promotes hepato-oncogenesis through inhibition of tumor-suppressive β-catenin/BAMBI signaling
Source: Exp Mol Med. 2016 Dec 2;48(12):e275–. doi: 10.1038/emm.2016.107 (PMC5192070; doi:10.1038/emm.2016.107)

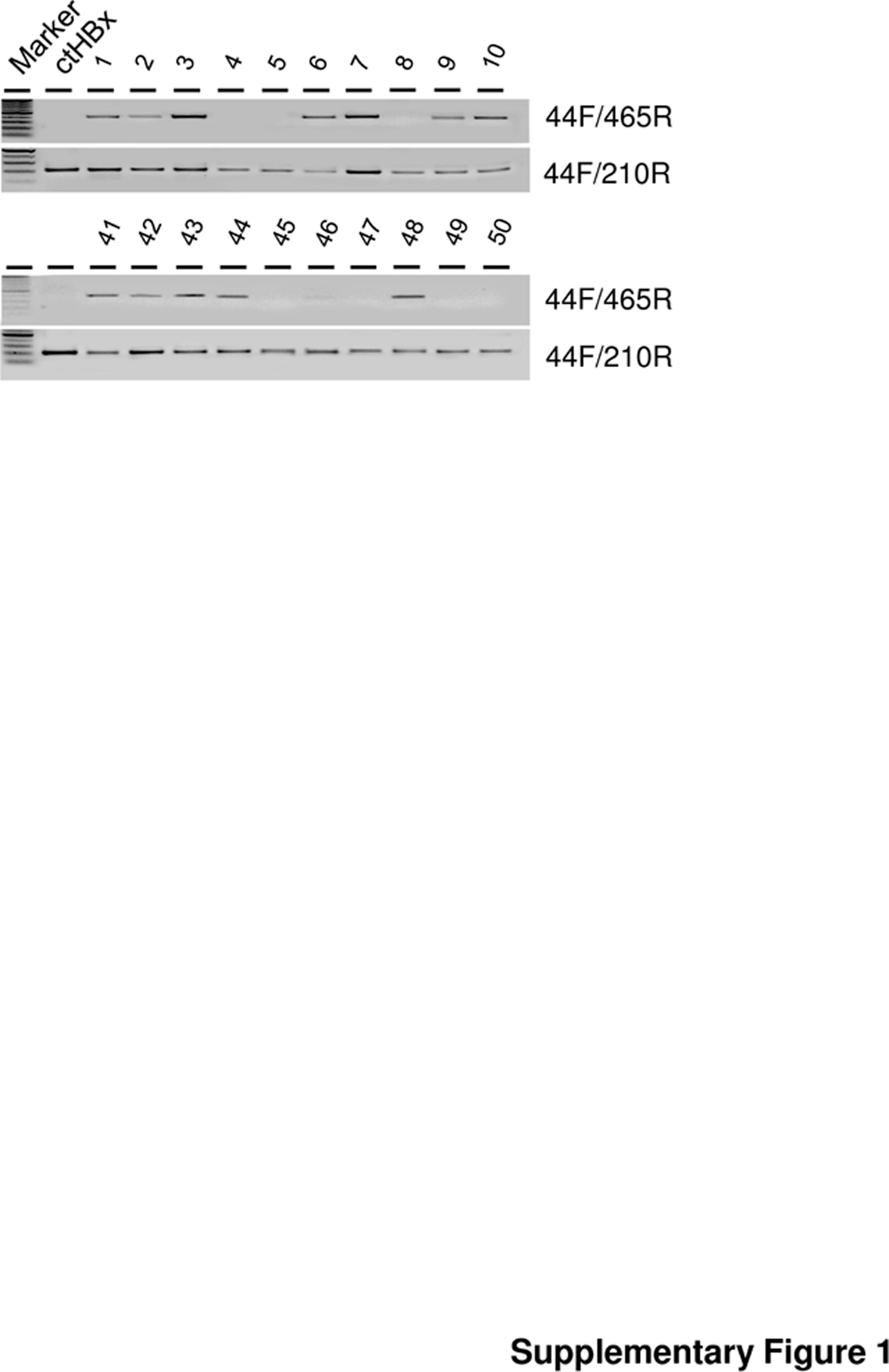

Supplement: Supplementary Figure 1 [file emm2016107x1.tif]

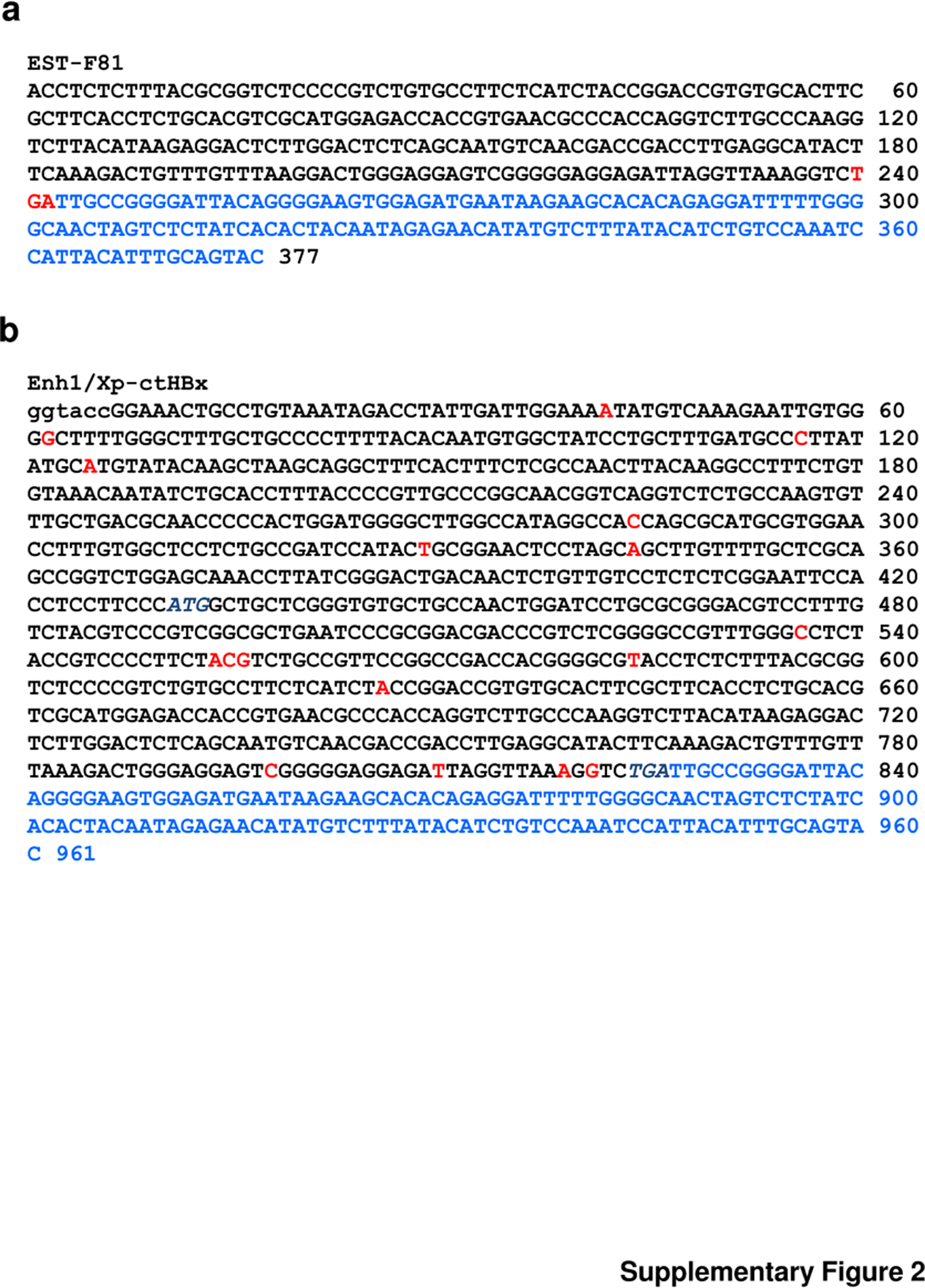

Supplement: Supplementary Figure 2 [file emm2016107x2.tif]

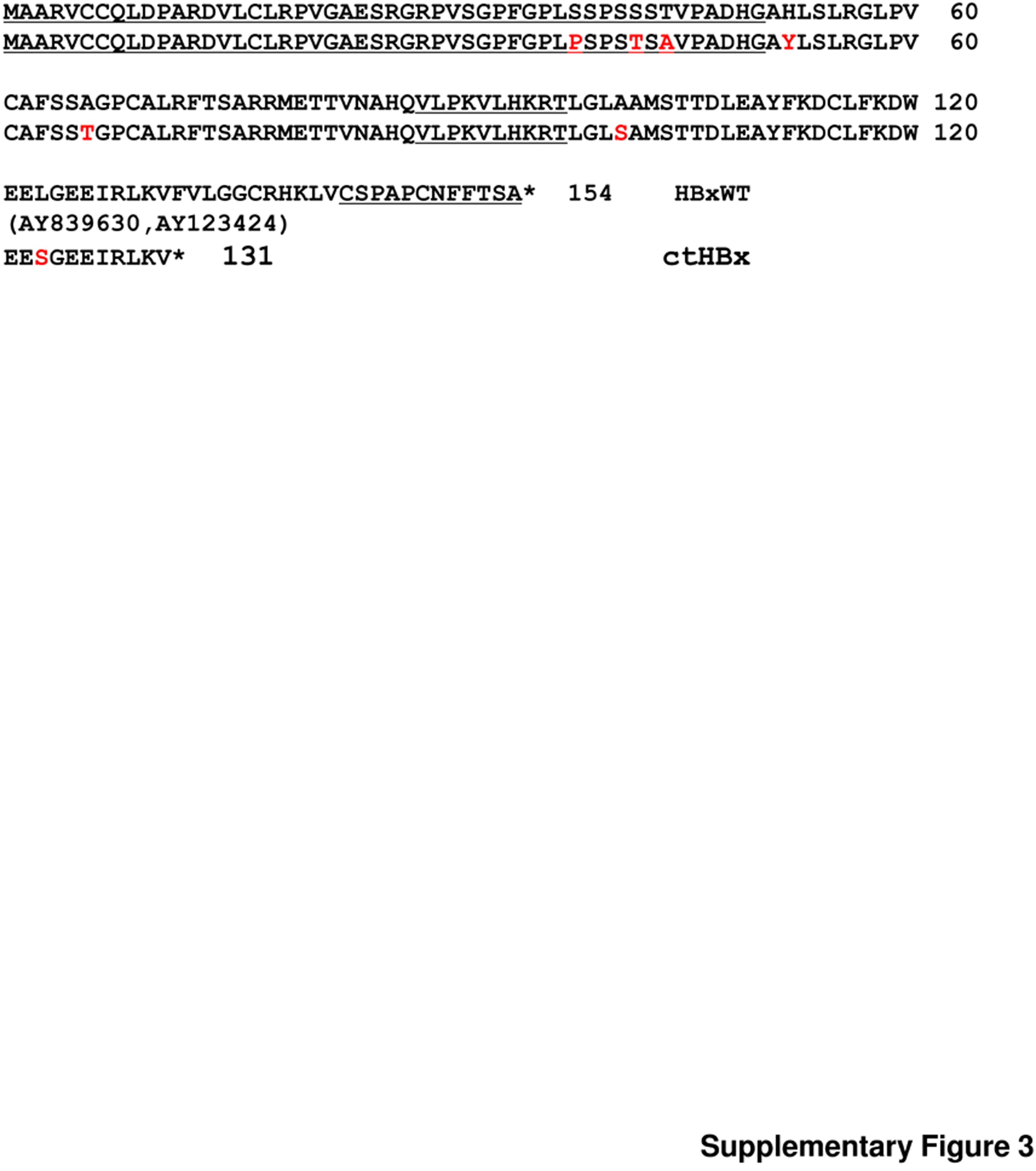

Supplement: Supplementary Figure 3 [file emm2016107x3.tif]

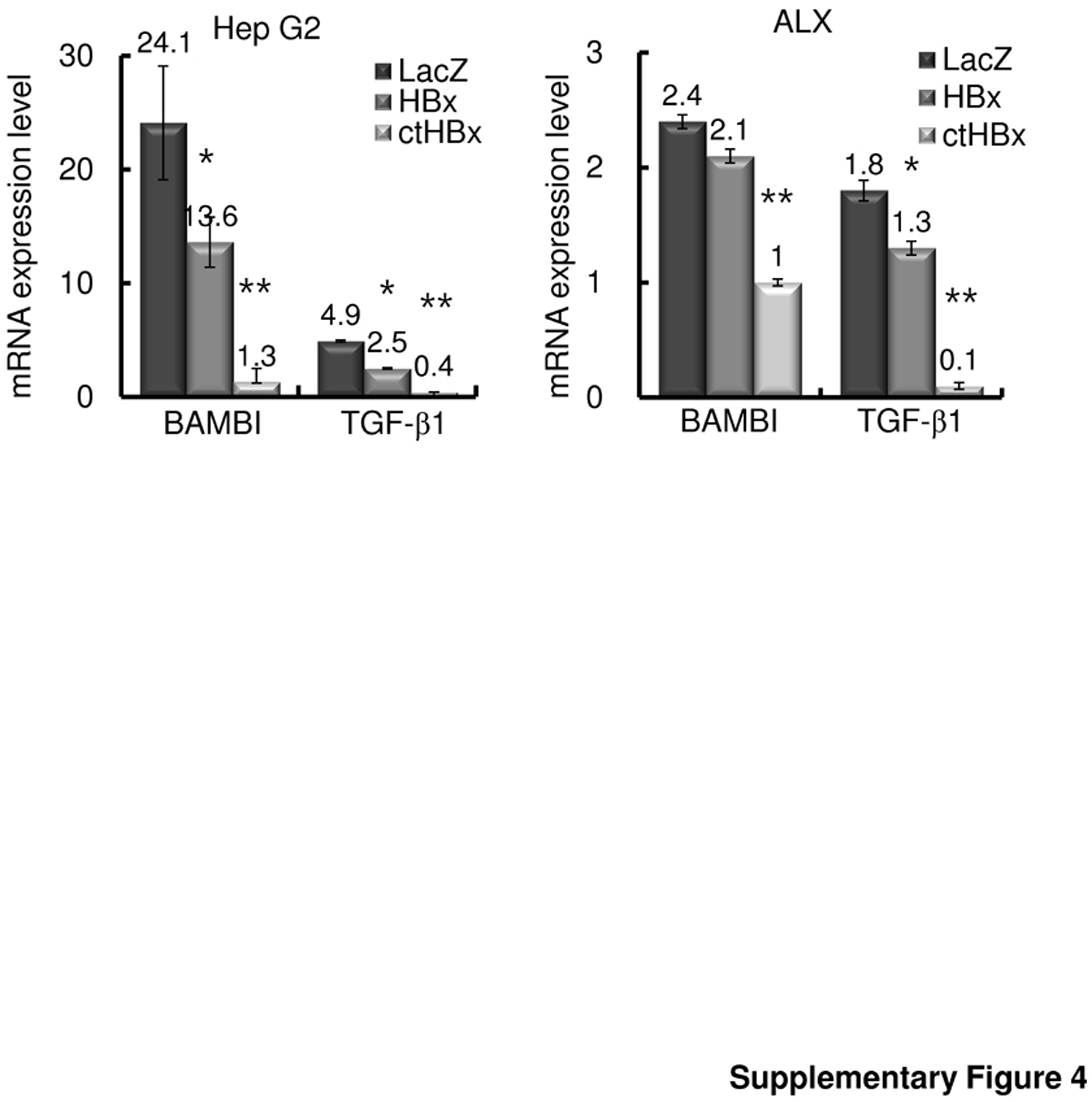

Supplement: Supplementary Figure 4 [file emm2016107x4.tif]

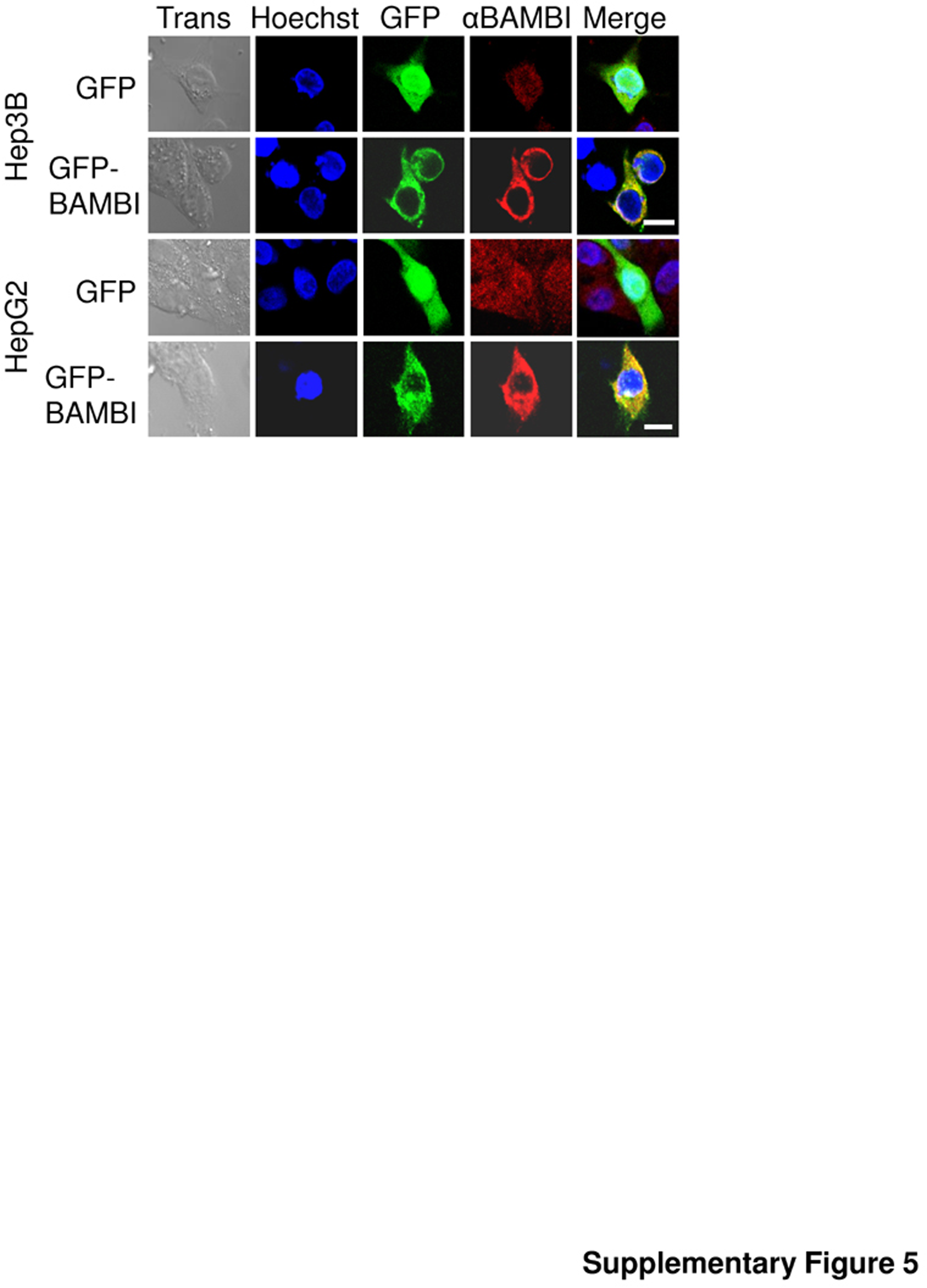

Supplement: Supplementary Figure 5 [file emm2016107x5.tif]

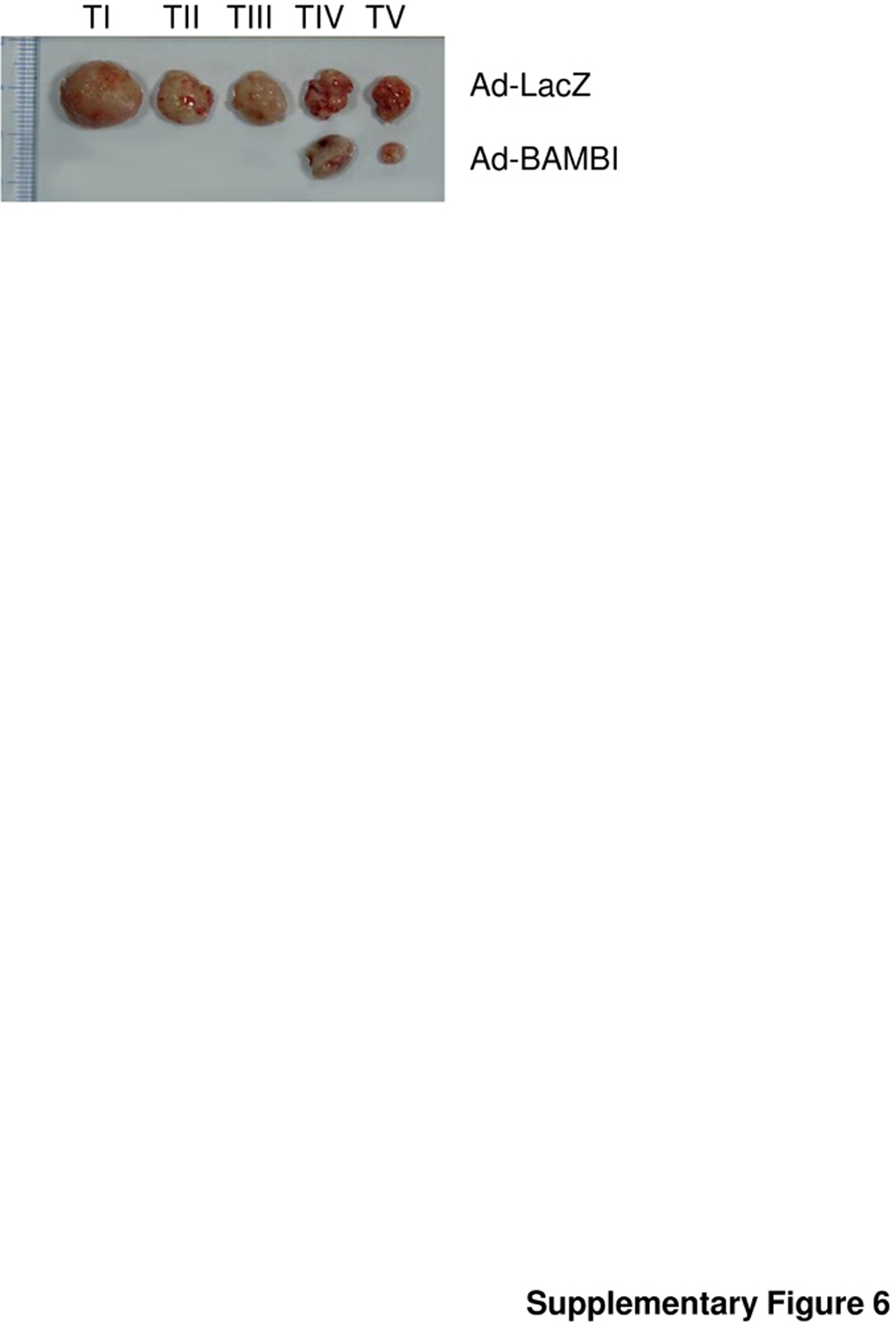

Supplement: Supplementary Figure 6 [file emm2016107x6.tif]
