# Supplementary material for: C-terminal-truncated HBV X promotes hepato-oncogenesis through inhibition of tumor-suppressive β-catenin/BAMBI signaling
Source: Exp Mol Med. 2016 Dec 2;48(12):e275–. doi: 10.1038/emm.2016.107 (PMC5192070; doi:10.1038/emm.2016.107)
Supplement: Supplementary Information [file emm2016107x7.docx]

**Supplementary information**

**C-terminal truncated HBV X promotes hepato-oncogenesis through inhibition of tumor-suppressive β-catenin/BAMBI signaling**

**Seok Lee^1,2,*^, Mi-Jin Lee^1,2,*^, Jun Zhang^1,2,*^, Goung-Ran Yu^1,2^ and Dae-Ghon Kim^1,2^**

^1^Division of Gastroenterology and Hepatology, Department of Internal Medicine, The Research Institute of Clinical Medicine of Chonbuk National University, Jeonju, Jeonbuk, Republic of Korea and ^2^Biomedical Research Institute of Chonbuk National University Hospital, Jeonju, Jeonbuk, Republic of Korea

Running Title: Suppression of BAMBI signaling by ctHBx

**^*^** These authors contributed equally to this work.

Correspondence: Dr D-G Kim, Division of Gastroenterology and Hepatology, Department of Internal Medicine, The Research Institute of Clinical Medicine of Chonbuk National University and Hospital, 20 Geonji-ro, Jeonju, Jeonbuk 54907, Republic of Korea

**RESULTS**

**Supplementary Table 1. Clinical characteristics of tumor and non-tumor tissues in two patients with HCC.**

| **Case** | **Age/Sex** | **Size**  **(cm)** | **Microvessel invasion** | **Survival**  **years** | **HBV**  **serology** | **Differentiation**  **(Edmondson)** | **AFP** | **Non-tumor tissue** |
| --- | --- | --- | --- | --- | --- | --- | --- | --- |
| 1 | 68/M | 3 × 2.5 | - | 5.4 | HBs Ag +  HBe Ab + | Grade II | 4.8 | Mild/moderate/cirrhosis (micronodular)* |
| 2 | 76/M | 7 × 5.8 | + | 1 | HBs Ab +  HBe Ab + | Grade I | 3.0 | None/none/none |

HBV, hepatitis B virus; AFP, alpha-feto protein

*Histologic activity of non-tumor tissue (grade and stage); periportal/lobular/fibrosis

**Supplementary Table 2. Isolation of genes with frequency in forward subtractive suppression hybridization (SSH) between ctHBx-associated HCC and non-tumor tissues.**

| **Accession No.** | **Description** | **Symbol** | **Frequency** |
| --- | --- | --- | --- |
| NM_002065 | Glutamate-ammonia ligase (glutamine synthase) | GLUL | 209 |
| DQ683578  L15533 | HBV X protein  Pancreatitis associated protein | HBX  PAP | 104  53 |
| NM_017460 | Cytochrome p450,subfamily IIIA,polypeptide 4 | CYP3A4 | 20 |
| NM_021107 | Mitochondrion (12S ribosomal protein) | MRPS12 | 14 |
| NM_014324 | Alpha-methylacyl-CoA racemase | AMACR | 11 |
| NM_021871 | Fibrinogen, A alpha polypeptide | FGA | 11 |
| NM_005888 | solute carrier family 25, member 3 | SLC25A3 | 10 |
| NM_012342 | BMP and activin membrane-bound inhibitor homolog | BAMBI | 9 |
| NM_000331 | Serum amyloid A | SAA1 | 9 |
| NM002954 | Mitochondrion (cytochrome b) |  | 8 |
| NM_020980 | Aquaporin 9 (AQP9), small solute channel 1 | AQP9 | 7 |
| NM_014495 | Angiopoietin-like 3 | ANGPTL3 | 6 |
| AK000796 | FLJ20789 fis | FLJ20789 fis | 6 |
| NM_003851 | Cellular repressor of E1A-stimulated genes | CREG | 5 |
| AK056736 | FLJ32174 fis | FLJ32174 fis | 5 |
| NM_000596 | IGFBP1 | IGFBP1 | 5 |
| AK055707 | FLJ31145 fis | FLJ31145 fis | 5 |
| NM_001128310 | SPARC-like 1 (mast9, hevin) | SPARCL1 | 5 |
| NM_001102470 | Alcohol dehydrogenase 6 (class V) | ADH6 | 4 |
| NM_005318 | H1-histone family,member 0 | H1F0 | 4 |
| AB049948 | Mitochondrion (16S ribosomal protein) | MRPS16 | 4 |
| X62996 | Mitochondrion (ATP synthase 6 + cytochrome c oxidase subunit III) |  | 4 |
| NM_005063 | Stearoyl-CoA desaturase | SCD | 4 |

**Supplementary Table 3. Isolation of genes with frequency in reverse suppression subtractive hybridization (SSH) between ctHBx-associated HCC and surrounding non-tumor tissues.**

| **Accession No.** | **Description** | **Symbol** | **Frequency** |
| --- | --- | --- | --- |
| NM_000477 | Albumin, | ALB | 81 |
| NM_000014 | Alpha-2-macroglobulin | A2M | 69 |
| NM_000035 | Aldolase B | ALDOB | 68 |
| NM_004467 | Dibrinogen like1, HCC related mRNA HFREF1 | FGL1 | 47 |
| NM_000596 | IGFBP1 | IGFBP1 | 34 |
| NM_005143 | Haptoglobin | HP | 27 |
| BC050388 | Similar to immunoglobulin lambda-like  polypeptide 1 |  | 22 |
| NM000990.1 | Complement component 9 | C9 | 21 |
| NM_006843 | Serine dehydratase | SDS | 17 |
| NM_001063 | Transferrin | TF | 15 |
| NM_021871 | Fibrinogen A alpha polypeptide | FGA | 14 |
| NM002954 | Fibrinogen gamma polypeptide | FGG | 10 |
| AF130077 | PRO2619 |  | 10 |
| K01562 | Ro RNA (scRNA) hY1 from small cytoplasmic ribonucleoprotein particles |  | 9 |
| NM000996.1 | ATP synthase, H+ transporting, mitochondrial F1 complex, alpha subunit, isoform 1, cardiac muscle | ATP5A1 | 8 |
| M11949 | Complement component 3 | C3 | 8 |
| NM_004417 | Dual specificity phosphatase 1 | DUSP1 | 8 |
| NM_002591 | Phosphoenolpyruvate carboxy kinase 1 | PCK1 | 8 |
| NM001012.1 | Decorin | DCN | 7 |
| NM_005141 | Fibrinogen,B beta polypeptide | FGB | 7 |
| XM_002345543 | Hypothetical protein LOC100293679 | LOC100293679 | 7 |
| NM_003725 | 3-Hydroxysteroid epimerase | RODH | 6 |
| NM_005518 | 3-Hydroxy-3-methylglutaryl-coenzyme A synthase 2 (mitochondrial) | HMGCS2 | 5 |
| NM_001831 | Clusterin | CLU | 5 |
| J04988 | Heart mRNA for heat shock protein 90 | HSP90 | 5 |
| NM_014216 | Inositol 1,3,4-triphosphate 5/6 kinase | ITPK1 | 5 |
| NM_019111 | Major histocompatibility complex, class II, DRA | HLA-DRA | 5 |
| NM_006169 | Nicotinamide N-methyltransferase | NNMT | 5 |
| NM_004666 | Vanin 1 | VNN1 | 5 |
| NM_000096 | Ceruloplasmin (feroxidase) | CP | 4 |
| NM_001735 | Complement component 5 | C5 | 4 |
| AF201940 | DC6 protein | DC6 | 4 |
| NM_000034 | Fructose-1,6-bisphosphatase 1 | FBP1 | 4 |
| NM_018487 | Hepatocellular carcinoma-associated antigen 112 | HCA112 | 4 |
| NM_000224 | Keratin 18 | KRT18 | 4 |
| NM_005807 | Proteoglycan4 | PRG4 | 4 |
| NM_000993 | Ribosomal protein L31 | RPL31 | 4 |
| AL360176 | RP11-124H7 on chromosome 10 | RP11-124H7 | 4 |
| NM_000295 | Serine (or cysteine) proteinase inhibitor, clade A, member 1 | SERPINA1 | 4 |
| NM_001085 | Serine (or cysteine) proteinase inhibitor, clade A, member 3 | SERPINA3 | 4 |
| AJ890084 | Tumor rejection antigen (gp96) 1 | TRA1 | 4 |

**Supplementary Figure Legends**

**Supplementary Figure 1** Detection of HBx with intact C-terminal (positive bands with 44F/465R primer set) and C-terminal truncated forms (positive bands with 44F/210R primer set and negative with 44F/465R primer set) of HBx DNA in HCC samples from patients with positive HB antigen serology.

**Supplementary Figure 2** The nucleotide and putative amino acid sequences of enhancer 1 and X promoter (*Enh1/Xp*) and the *ctHBx* of HBV cDNA. (**a**) The nucleotide sequence of the expressed-sequence tag (EST) clone F81, which contained a partial HBx (black letters) with a stop codon (red letters) and human chromosome 8 genomic contig sequences (blue letters). (**b**) The nucleotide sequences of enhancer 1/X promoter and the ctHBx (*Enh1/Xp-ctHBx*) from the HBV cDNA. Red letters represent mutated nucleotides.

**Supplementary Figure 3** Putative amino acids sequences of the *ctHBx* (lower row) compared to those of wild-type *HBx* (upper row). Underlined domains represent the transforming domain (aa 1―50), the p53-mediated repression domain (aa 88―97), and growth-suppressive effect domain (aa142―154), in that order. Red letters represent mutated amino acids.

**Supplementary Figure 4** mRNA expression levels of BAMBI and TGF-β1 in HepG2 or ALX cells transiently transfected with ctHBx or HBx expression plasmids.

**Supplementary Figure 5** Subcellular localization of the BAMBI protein. Hep3B and HepG2 cells were transiently transfected with GFP-tagged BAMBI, fixed in 0.4% paraformaldehyde, and subsequently processed for indirect immunofluorescence with an antibody against BAMBI (TRITC, red). The nuclei were stained with Hoechst 33258 (blue), and the cells were subsequently examined using confocal microscopy (Trans, transmission; bar, 20 µm).

**Supplementary Figure 6** Gross appearance of the tumor masses in the SH-J1 cells infected with either Ad-LacZ or Ad-BAMBI.
